# Supplementary material for: Drug repurposing for aging research using model organisms
Source: Aging Cell. 2017 Jun 16;16(5):1006–15. doi: 10.1111/acel.12626 (PMC5595691; doi:10.1111/acel.12626)
Supplement: Supplementary file 7 — Data S1 Zip‐Archive of all report cards. [file ACEL-16-1006-s007.zip › RC_GOA.pdf]

GOA

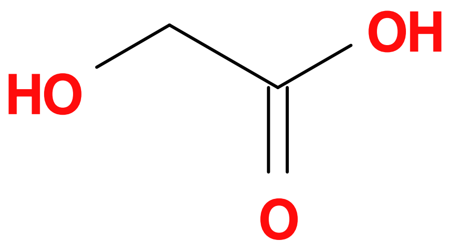

Database identifiers

ChEMBLCompound	CHEMBL252557
DrugBank	DB03085
CHEBI	17497
eMolecules	479771

Ranking

	Rank	Score
Drosophila	501/697	0.272
C. elegans	291/591	0.197

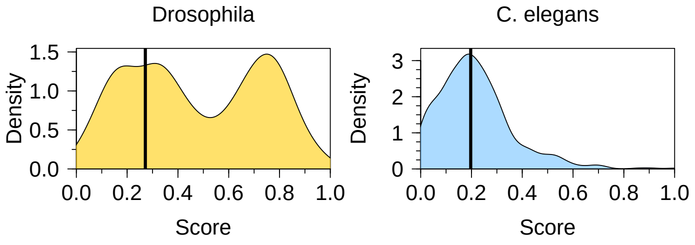

	Ageing implication	Domain conservation	Binding site conservation	Binding affinity	Bioavailability	Lipinski	Promiscuity	Purchasability	Drug approval	**Total**
Drosophila	0.36	0.938	1.0	0.32	(0.9)	0.0	-0.0	0.1	0.075	0.272
C. elegans	0.36	0.914	1.0	0.32	0.214	0.0	-0.0	0.1	0.075	0.197

Names

- **glycolic acid**
- alpha-hydroxyacetic acid
- Hoch2cooh
- Hydroxyacetic Acid
- Hydroxyethanoic acid

Roles

metabolite, keratolytic drug

Status

Approved drug (according to ChEMBL)	No
Classification (according to DrugBank)	experimental
Number of Rule of 5 violations	0
Binding affinity to original target in log units (RF-Score prediction)	4.24
Burns *C. elegans* bioavailability prediction	-5.07

Compound Target Characteristics

Glucose-6-phosphate 1-dehydrogenase

Best gene implication in ageing for this target family came from gene P12646 via mapping the annotation from Ensembl FBgn0004057 via mapping the annotation from EntrezGene 32974 via mapping the annotation from GenAgeModels 0883 annotated in GenAge release 17.

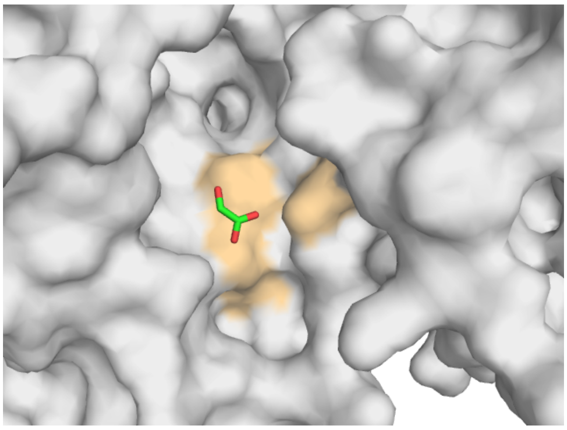

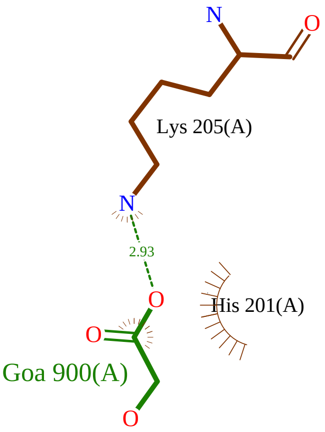

protein	amino acids contacts (binding site)
PDB:1qki:chainA:P11413	K H K
tr:E9PD92:E9PD92_HUMAN	K H K
tr:E7EM57:E7EM57_HUMAN	K H K
tr:E7EUI8:E7EUI8_HUMAN	K H K
tr:Q2VF42:Q2VF42_HUMAN	K H K
sp:P11413:G6PD_HUMAN	K H K
sp:P05370:G6PD_RAT	K H K
tr:A3KG36:A3KG36_MOUSE	K H K
tr:Q790Y8:Q790Y8_MOUSE	K H K
sp:Q00612:G6PD1_MOUSE	K H K
tr:G3UWD6:G3UWD6_MOUSE	K H K
sp:P12646:G6PD_DROME	K H K
tr:B7FNK0:B7FNK0_DROME	K H K
sp:Q27464:G6PD_CAEEL	K H K
sp:P11412:G6PD_YEAST	K H K

protein	whole protein ident	simil	domain-based ident	simil	contact-based ident	simil
PDB:1qki:chainA:P11413	1.0	1.0	1.0	1.0	1.0	1.0
tr:E9PD92:E9PD92_HUMAN	0.5	0.5	1.0	1.0	1.0	1.0
tr:E7EM57:E7EM57_HUMAN	0.62	0.62	0.99	0.99	1.0	1.0
tr:E7EUI8:E7EUI8_HUMAN	0.65	0.65	0.99	0.99	1.0	1.0
tr:Q2VF42:Q2VF42_HUMAN	0.92	0.92	0.94	0.94	1.0	1.0
sp:P11413:G6PD_HUMAN	1.0	1.0	1.0	1.0	1.0	1.0
sp:P05370:G6PD_RAT	0.94	0.98	0.91	0.97	1.0	1.0
tr:A3KG36:A3KG36_MOUSE	0.71	0.75	0.92	0.97	1.0	1.0
tr:Q790Y8:Q790Y8_MOUSE	0.94	0.98	0.92	0.98	1.0	1.0
sp:Q00612:G6PD1_MOUSE	0.94	0.98	0.92	0.98	1.0	1.0
tr:G3UWD6:G3UWD6_MOUSE	0.87	0.96	0.86	0.96	1.0	1.0
sp:P12646:G6PD_DROME	0.61	0.85	0.59	0.87	1.0	1.0
tr:B7FNK0:B7FNK0_DROME	0.61	0.85	0.59	0.87	1.0	1.0
sp:Q27464:G6PD_CAEEL	0.57	0.83	0.54	0.84	1.0	1.0
sp:P11412:G6PD_YEAST	0.44	0.77	0.42	0.79	1.0	1.0

Zw (FBgn0004057) associated phenotypes

long lived, oxidative stress response defective

(Information from FlyBase)

Zw (UniProt:P12646) annotation

Function: Catalyzes the rate-limiting step of the oxidative pentose-phosphate pathway, which represents a route for the dissimilation of carbohydrates besides glycolysis. The main function of this enzyme is to provide reducing power (NADPH) and pentose phosphates for fatty acid and nucleic acid synthesis (By similarity).
Pathway: Carbohydrate degradation; pentose phosphate pathway; D- ribulose 5-phosphate from D-glucose 6-phosphate (oxidative stage): step 1/3.

(Information from UniProt)

Zw-RA (UniProt:B7FNK0) annotation

Pathway: Carbohydrate degradation; pentose phosphate pathway; D- ribulose 5-phosphate from D-glucose 6-phosphate (oxidative stage): step 1/3. (RuleBase:RU000497).

(Information from UniProt)

gspd-1 (WBGene00007108) associated phenotypes

lethal, sterile

(Information from WormBase)

gspd-1 (UniProt:Q27464) annotation

Function: Catalyzes the rate-limiting step of the oxidative pentose-phosphate pathway, which represents a route for the dissimilation of carbohydrates besides glycolysis. The main function of this enzyme is to provide reducing power (NADPH) and pentose phosphates for fatty acid and nucleic acid synthesis (By similarity).
Pathway: Carbohydrate degradation; pentose phosphate pathway; D- ribulose 5-phosphate from D-glucose 6-phosphate (oxidative stage): step 1/3.

(Information from UniProt)
